# The Audiological Aspect of Beckwith–Wiedemann Syndrome: A Systematic Review

**DOI:** 10.3390/genes17040453

**Published:** 2026-04-14

**Authors:** Sara Parretta, Michele Pellegrino, Laura Luppi, Elena Braglia, Elisabetta Genovese, Davide Soloperto

**Affiliations:** Department of Otolaryngology, Head and Neck Surgery, University Hospital of Modena, 41125 Modena, Italy; 342805@studenti.unimore.it (S.P.); 83118@studenti.unimore.it (L.L.); 188313@studenti.unimore.it (E.B.); elisabetta.genovese@unimore.it (E.G.); davide.soloperto@unimore.it (D.S.)

**Keywords:** Beckwith–Wiedemann syndrome, hearing loss, stapes fixation, cochlear implant, audiological rehabilitation

## Abstract

**Background**: Beckwith–Wiedemann syndrome (BWS) is a rare congenital overgrowth disorder caused by genetic and epigenetic alterations on chromosome 11p15.5. While macroglossia, abdominal wall defects, and tumor predisposition are well recognized, hearing impairment has been sporadically reported. **Objectives**: The aim of this study is to review audiological features, surgical management, and rehabilitation in BWS, and we additionally present three cases with comprehensive longitudinal audiological follow-up. **Methods**: A systematic review of PubMed and Scopus was conducted according to PRISMA guidelines, including studies reporting audiological findings in patients with confirmed BWS. Studies without audiological data or reporting only normal-hearing patients were excluded. Data on hearing loss type, severity, genetics, clinical features, imaging, surgical interventions, and outcomes were extracted. A narrative synthesis was conducted; no meta-analysis was performed due to the heterogeneity and limited number of available studies. Data extraction was performed independently by two reviewers who independently screened titles, abstracts, and full texts, with disagreements resolved by discussion. In addition, three original case reports from our institution were included to further illustrate the clinical and rehabilitative variability of hearing impairment in BWS. **Results**: We identified 40 patients from the review, but only 12 of them reported audiological data (e.g., hearing thresholds, type of hearing loss, or diagnostic tests). Ossicular chain anomalies, particularly stapes fixation, were frequently observed. Surgical management improved hearing in selected cases, while bone conduction devices (BCD) or conventional amplification were effective alternatives when surgery was contraindicated. Genetic analyses revealed CDKN1C mutations or imprinting defects in nine patients. **Conclusions**: Hearing impairment in BWS is clinically relevant and often conductive, likely related to middle-ear anomalies. Early, multidisciplinary audiological evaluation—including imaging when indicated—and individualized rehabilitation can optimize auditory and communicative outcomes. The evidence is limited by the small number of studies and heterogeneous reporting of audiological outcomes.

## 1. Introduction

Beckwith–Wiedemann syndrome (BWS) is a congenital overgrowth disorder characterized by macroglossia, gigantism, abdominal wall defects and an increased risk of tumor development [1]. It is a rare imprinting disorder, with an estimated prevalence of 1–5 per 10,000 live births, and occurs with equal frequency in males and females, except among monozygotic twins, who exhibit a marked female predominance. Most cases arise sporadically (approximately 85%), while familiar transmission occurs for the remaining 15%.

BWS is associated with a wide spectrum of genetic and epigenetic alterations, primarily affecting growth-regulating genes located on chromosome 11p15.5, which contribute to clinical heterogeneity. Common clinical features, which are classically stratified into major and minor diagnostic criteria and are utilized to establish a clinical diagnosis [2] (Table 1), include increased growth parameters (macrosomia, hemihypertrophy), abdominal wall defects (such as omphalocele or umbilical hernia), macroglossia, visceromegaly, a predisposition to embryonal tumors (particularly Wilms tumor and hepatoblastoma), postnatal hypoglycemia, anterior ear lobe creases and/or posterior helical pits, as well as renal abnormalities. Auditory impairments have also been reported in the literature in association with Beckwith–Wiedemann syndrome, particularly hearing loss related to anomalies of the middle ear ossicular chain, including stapes fixation and incudostapedial malformations [3].

Despite several case reports and small series describing hearing impairment in Beckwith–Wiedemann syndrome, the available evidence is extremely limited, heterogeneous, and lacks a systematic synthesis. No comprehensive systematic review has previously summarized the audiological phenotype, associated otologic anomalies, and management strategies in BWS. Therefore, a systematic evaluation of the existing literature was needed to clarify the prevalence, characteristics, and clinical implications of hearing loss in this population, and to support clinicians in diagnostic and rehabilitative decision-making.

The aim of this review is to investigate and describe the audiological features, surgical treatments and possible hearing rehabilitation in patients affected by BWS.

In addition, we present three original clinical cases from our institution to illustrate the heterogeneity of audiological presentation and longitudinal management in BWS.

## 2. Materials and Methods

**Figure 1:**
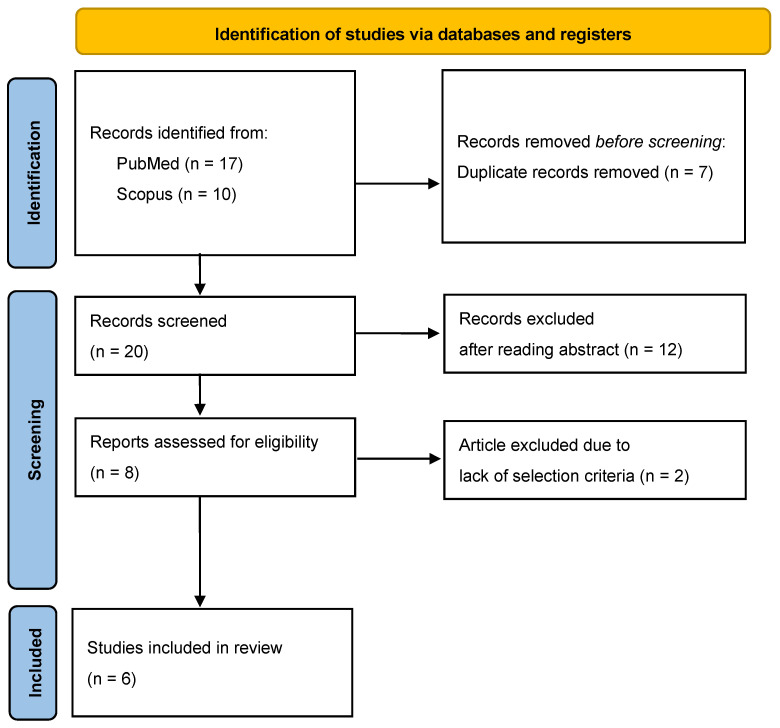
PRISMA 2020 flow diagram of study selection.

This systematic review was conducted and reported in accordance with the PRISMA 2020 statement. The review protocol was not registered in PROSPERO or in any other database. A PRISMA flow diagram summarizing the study selection process is included in the manuscript (Figure 1).

A comprehensive literature search was performed in PubMed and Scopus with the following search string: (“Beckwith-Wiedemann Syndrome” OR “Beckwith-Wiedemann syndrome” OR “BWS”) AND (“hearing loss” OR “audiological” OR “hearing aids” OR “cochlear implant” OR “ENT”); no filters (language, date, or study type) were applied.

No additional databases, registers, websites, or gray literature sources were consulted. The last search of all sources was performed on 10 February 2026.

Studies were considered eligible if they were published in the English language, included patients with a confirmed diagnosis of BWS, and provided a description of audiological data (e.g., type/degree of hearing loss, laterality, thresholds, results of audiological tests, or relevant otologic findings). When available, details regarding the type and degree of hearing loss as well as information on surgical and/or prosthetic hearing rehabilitation were also required. Studies lacking audiological assessment or reporting exclusively normoacusic patients were excluded.

No restrictions regarding the year of publication were applied due to the limited amount of available literature on the otorhinolaryngological and audiological manifestations of BWS. This choice was made because the available literature on audiological and otologic manifestations in BWS is extremely limited and sporadically distributed over several decades; indeed, the scientific evidence addressing these aspects is scarce, with approximately three publications per two-decade period. Notably, three articles were published between 1980 and 2000, and a comparable number between 2001 and 2020, highlighting the persistent paucity of research in this field. For this reason, applying a temporal restriction would have risked excluding relevant data.

The study selection and data extraction processes were performed by two reviewers who independently screened all titles and abstracts and compiled a predefined data extraction form. The full texts of potentially eligible articles were assessed independently by the same reviewers. Disagreements were resolved through discussion. For each included study, both reviewers extracted information on study characteristics, audiological findings, genetic results, imaging, surgical interventions, and rehabilitation outcomes. No assumptions were made for missing data; unreported variables were recorded as not available. All extracted data were cross-checked, and discrepancies were resolved through discussion. Authors were not contacted and no automated tools were used.

Since this review synthesized heterogeneous case reports and case series without performing a meta-analysis, no predefined effect measures were applied, nor was a formal assessment of reporting bias applicable.

Given the heterogeneity and the descriptive nature of the evidence, we conducted a narrative synthesis. Studies were grouped and summarized by hearing loss type (conductive, sensorineural, or mixed), degree, laterality, and key audiological or surgical findings; results were tabulated when possible. No quantitative synthesis, subgroup analyses, or sensitivity analyses were planned or performed, and no statistical heterogeneity metrics were computed. This approach aligns with the absence of predefined effect measures and the non-feasibility of a meta-analysis.

No formal assessment of certainty (e.g., GRADE) was performed because the included evidence derived from heterogeneous case reports and case series, for which such evaluation is not applicable.

## 3. Results

The initial search yielded a total of 27 records (17 from PubMed and 10 from Scopus). After the removal of seven duplicate articles, 20 studies underwent title and abstract screening, resulting in the exclusion of 12 records. Subsequently, eight full-text articles were assessed for eligibility. Among these, six studies describing patients with hearing loss in the context of syndromic BWS met the inclusion criteria and were included in the final qualitative synthesis. The included studies showed generally low methodological robustness due to the inherent limitations of case reports and case series, but all provided sufficient detail to support inclusion.

Because no meta-analysis or quantitative synthesis was conducted, no assessment of reporting bias was applicable.

### 3.1. Population

A total of 40 patients diagnosed with Beckwith–Wiedemann syndrome (BWS) were identified through the systematic review. Among these, hearing loss was reported in 12 patients. All subsequent analyses were therefore conducted exclusively on this subgroup of patients with documented hearing impairment.

The 12 patients with hearing loss included four females, four males, and four patients with unspecified sex. Audiometric evaluation or age at assessment was available for 10 patients (mean age: 6.16 years). In the remaining two patients, although audiometric and chronological details were not available, hearing loss was clearly described by the original authors and supported by the reported otologic management. Given the limited literature on BWS, these cases were retained.

### 3.2. Audiological Characteristics

Hearing loss severity was classified according to the criteria reported in the Camet M.L. et al. study [4] (0–20 dB HL: normal; 21–40: mild; 41–70: moderate; >70: severe/profound). This represents an operational classification used by the authors and does not correspond to any specific internationally recognized audiological classification (e.g., WHO, BIAP, or ASHA), as it differs in threshold cut-offs and category grouping; therefore, in order to ensure consistency and allow comparison across all included studies, the same classification was applied uniformly throughout the present review.

Among the 12 patients with hearing loss, four were diagnosed with sensorineural (SNHL)/mixed hearing loss and eight with conductive hearing loss (CHL).

Among the patients with sensorineural/mixed HL (*n* = 4): one patient had mild bilateral SNHL; two patients had severe/profound bilateral sensorineural/mixed; and one patient had moderate bilateral SNHL.

Among the patients with CHL (*n* = 8): two patients presented with unilateral mild CHL (one right-sided and one left-sided); and six patients had bilateral CHL, although the degree of hearing loss was not specified (Table 2).

### 3.3. Genetic Findings

Genetic data was not available for all patients with hearing loss (*n* = 12). Genetic confirmation of BWS was reported in nine patients. Specifically: three patients carried a CDKN1C mutation c.579delT (p.A193AfsX46); three patients carried a CDKN1C mutation c.881delC (p.Pro294fs); one patient showed hypermethylation of imprinting center 1 (IC1); one patient showed hypermethylation of imprinting center 2 (IC2); and in one patient, genetic analysis did not reveal any detectable molecular abnormalities and the diagnosis of BWS was based on the clinical findings, including the craniofacial, otologic, gastrointestinal, dermatologic, or developmental features [7]. In the remaining three patients, no information regarding genetic evaluation or diagnosis methodology was reported (Table 2).

### 3.4. Clinical Characteristics

Macroglossia was reported in five patients, cleft palate in three patients, omphalocele in five patients, and flame nevus in two patients. A language disorder was reported in one patient, described in the context of associated intellectual disability. Craniofacial anomalies, including midfacial hypoplasia, high-arched palate, prognathism, and anterior open bite, were reported in four patients.

External ear malformations were described in six patients. Specifically: earlobe sulci were observed in two patients; earlobe indentation was reported in four patients; and anterior ear creases and indent lesions on the posterior rim of the helix were described in three patients, all of whom also presented with earlobe indentation. Thus, three patients exhibited more than one auricular malformation [5].

Otoscopy (visual inspection of the external auditory canal and tympanic membrane) was reported in five of the six patients with external ear anomalies and consistently demonstrated normal findings. In the remaining patients, neither otoscopic findings nor other clinical features typically associated with BWS were described (Table 3).

### 3.5. Imaging

Computed tomography (CT) of the temporal bone was performed in only one patient, revealing bilateral anterior bony fixation of the malleus in the epitympanum [7].

### 3.6. Surgery

Only five patients underwent exploratory tympanotomy. In four patients (one of whom underwent unilateral right-sided exploratory tympanotomy), intraoperative findings confirmed bony fixation of both mallei in the epitympanum and normally shaped but ankylotic stapes. Malleus fixation was managed by careful drilling, followed by small-fenestra stapedectomy. Stapedial fixation was confirmed intraoperatively, and satisfactory postoperative hearing improvement was reported.

We reported one patient underwent left-sided exploratory tympanotomy [5], which revealed total stapes fixation due to the absence of the ligament, along with a small pearl-like cholesteatoma located between the long process of the incus and the handle of the malleus. In addition, one patient underwent placement of tympanostomy drainage tubes (TTs); these patients correspond to a severe/profound HL [4].

Postoperative hearing improvement was reported in two patients: in one case, conductive hearing loss (CHL) improved to approximately 20 dB on the right side and 40 dB on the left side; in another case, progressive left-sided CHL stabilized, while right-sided thresholds improved from 70 dB with a 30 dB air–bone gap to postoperative thresholds of 30–35 dB (Table 4).

### 3.7. Hearing Rehabilitation

Among patients with sensorineural hearing loss (*n* = 4), three underwent hearing aid fitting. No patient underwent cochlear implantation.

### 3.8. Case Presentation

In this context, we report three additional patients with Beckwith–Wiedemann syndrome (BWS) followed at our institution, for whom comprehensive audiological evaluations and longitudinal follow-ups were available. The detailed description of these cases aims to further contribute to the characterization of the audiological phenotype in BWS and to illustrate different diagnostic and rehabilitative pathways within this patient population (Table 5).

#### 3.8.1. Case 1

The first patient, a female, was born as a late preterm (34 weeks) infant from a dichorionic diamniotic twin pregnancy by cesarean section and experienced a severe neonatal course, characterized by respiratory distress requiring invasive ventilatory support and complicated by necrotizing enterocolitis (NEC) necessitating surgical intervention.

Due to the presence of severe congenital macroglossia, characterized by a persistently protruding and poorly mobile tongue, in association with abnormal umbilical cord insertion, Beckwith–Wiedemann syndrome (BWS) was clinically suspected. The diagnosis was subsequently confirmed by molecular genetic analysis, which demonstrated the hypomethylation of the KvDMR locus (IC2 region, 11p15.5). At birth, transient evoked otoacoustic emissions (TEOAEs, sounds generated by outer hair cells in response to transient stimuli, used to assess cochlear function) were present bilaterally, whereas an automated auditory brainstem response (AABR, an automated electrophysiological screening test that evaluates auditory nerve and brainstem responses to sound stimuli) screening yielded a bilateral “refer” result.

The patient was first evaluated at our institution at 9 months of age, when a comprehensive audiological assessment was performed. An otoscopic examination revealed bilaterally normal external auditory canals and intact tympanic membranes. Flexible nasopharyngolaryngoscopy demonstrated an age-appropriate laryngeal morphology, a normotrophic tongue base, and the absence of laryngomalacia. Auditory brainstem response (ABR) threshold testing failed to elicit a reproducible wave V bilaterally, even at maximum stimulation levels (100 dB nHL), consistent with profound bilateral sensorineural hearing loss. Immittance audiometry demonstrated type A tympanograms, with absent acoustic stapedius reflexes bilaterally.

Based on the audiological findings, early bilateral hearing aid fitting was initiated as part of the pre-cochlear implantation assessment and rehabilitation pathway. Behavioral audiometry, performed both unaided and aided, failed to elicit reliable auditory responses, even at maximum stimulus intensity levels. Based on these results, further neuroradiological evaluation was requested to assess the integrity of the cochlear nerves and inner ear structures, as part of the cochlear implant candidacy work-up.

A contrast-enhanced magnetic resonance imaging (MRI) of the posterior cranial fossa demonstrated normal visualization of the cochlear nerves and facial–vestibulocochlear nerve bundles, as well as normal signal intensity of the cochleo-vestibular membranous labyrinths. High-resolution computed tomography (CT) of the temporal bones revealed normal external auditory canal caliber, well-aerated middle ear cavities, intact ossicular chains, normal internal auditory canal dimensions, and normal cochlear and vestibular morphology, with prominent semicircular canals, particularly the superior semicircular canals.

Based on the comprehensive audiological evaluation, behavioral assessment, and neuroradiological findings, cochlear implantation was confirmed as the appropriate intervention. The patient subsequently underwent simultaneous bilateral cochlear implantation using a Cochlear Nucleus CI612 at approximately 15 months of age. The surgical procedure was completed without intraoperative complications, and the postoperative course was uneventful, with no early or late surgical complications observed.

Cochlear implant processor activation was performed 15 days after the surgical procedure using a CP1110 processor. In the initial post-activation phase, the patient demonstrated auditory awareness only to high-intensity acoustic stimuli, and behavioral audiometry showed consistent responses limited to 2000 Hz at 80 dB HL.

At three months post-activation, aided free-field thresholds with bilateral cochlear implants showed a bilateral pure-tone average (PTA) of approximately 50 dB HL. Increased vocalizations and babbling, characterized by persistent tongue protrusion, were observed. From a communicative standpoint, the child demonstrated use of deictic gestures but did not consistently respond to her own name.

At six months post-activation, further improvement in auditory perception and behavioral responses was observed: the patient responded consistently to her own name, detected low-intensity stimuli in quiet listening conditions, and structured auditory assessments (Ambo 2.0 and SixSound tests) indicated emerging sound recognition. Babbling was present but still affected by tongue protrusion, and discrimination of familiar voices was not yet observed. The patient remains under ongoing audiological follow-up.

#### 3.8.2. Case 2

The second patient, a male, was born preterm (27 weeks) via cesarean section due to placenta previa. Newborn hearing screening at birth was normal bilaterally. A diagnosis of Beckwith–Wiedemann syndrome (BWS) had been previously established; however, the genetic documentation confirming the molecular subtype was not available, and the specific variant could not be verified. Clinically, the diagnosis was supported by several characteristic features, including macrosomia, omphalocele, and a facial nevus flammeus.

Right-sided conductive hearing loss was first identified at the age of 5 years.

At 6 years of age, the patient underwent right endoscopic exploratory tympanotomy at another institution. Intraoperatively, an abnormality of the incudostapedial joint was observed: the incus did not articulate with the malformed stapes, and a dehiscence of the facial nerve canal was noted. A careful removal of adhesions was performed, and a partial ossicular replacement prosthesis (PORP) was implanted between the anomalous stapes head and the handle of the malleus. The postoperative course was uneventful; however, no improvement in audiometric thresholds was achieved.

At 16 years of age, the patient was first evaluated at our center. Otoscopic examination revealed a normal tympanic membrane on the left, while the right ear showed regular post-ossiculoplasty findings with a dry neotympanum. An audiological assessment confirmed severe right-sided conductive hearing loss with a significant air–bone gap (average of 48.75 dB) and absent acoustic reflexes. Speech audiometry demonstrated a maximum word recognition score of 60% at 90 dB HL. Hearing in the left ear remained normal.

Given the current audiological profile and the normal contralateral hearing, surgical stapedial fenestration with a malleus–stapes prosthesis was contraindicated due to the ossicular malformation. Hearing rehabilitation with a bone conduction device (BCD) was therefore indicated. The patient subsequently underwent a trial with a BAHA 6 in softband configuration, which resulted in significant functional improvement in both hearing thresholds and speech perception, with a word recognition score of 100% at 70 dB HL. Based on these positive outcomes, the indication for the implantation of a bone-anchored hearing device was confirmed.

The patient is currently on the waiting list for the surgical procedure and continues to be followed at our center for ongoing audiological monitoring and rehabilitation.

#### 3.8.3. Case 3

The third patient, a female, was referred to our center for audiological evaluation after a newborn hearing screening yielded a “refer” result on transient evoked otoacoustic emissions (TEOAEs). She had a previously established diagnosis of Beckwith–Wiedemann syndrome (BWS), although the genetic documentation was not available to our team. However, the clinical presentation was strongly suggestive of BWS, as the patient exhibited several characteristic features including macrosomia, macroglossia, cleft palate, hemifacial hyperplasia, and multiple auricular fistulae. Her clinical history was notable for percutaneous endoscopic gastrostomy (PEG) and tracheostomy.

Audiological assessments, including TEOAEs and auditory brainstem response (ABR) testing, confirmed a bilateral hearing impairment of moderate degree, as verified on repeat testing. By the age of 5 years, binaural hearing thresholds were obtained, revealing bilateral conductive hearing loss with a mean PTA of 40 dB and an air–bone gap of about 35 dB; bilateral amplification was indicated. Previous cleft palate surgery outcomes were stable, although nasal reflux persisted.

During follow-up, the patient underwent regular assessments including pure-tone audiometry, aided testing with hearing devices, and evaluation of auditory perceptual skills, which were partially limited by coexisting language delay. The patient remains under regular audiological follow-up, continues to attend school, and is receiving appropriate amplification and rehabilitative support to optimize auditory and communicative development.

In light of the progression of hearing loss in the left ear and the worsening of the conductive component, a high-resolution computed tomography (HRCT) scan of the temporal bone was recommended to better characterize the middle ear structures and to further investigate the etiology of the conductive hearing loss.

## 4. Discussion

Across the studies included in this systematic review, hearing impairment was reported in approximately one-third of patients with Beckwith–Wiedemann syndrome (BWS). Although auditory involvement is not considered a core clinical feature of the syndrome, this finding suggests that audiological abnormalities may represent a clinically relevant, yet likely under-recognized, comorbidity. These data support the potential value of routine audiological surveillance in patients with BWS, particularly during early childhood, when adequate auditory input is essential for speech and language development. The available evidence does not indicate a sex-related predisposition to hearing loss. The overall balanced distribution between males and females is consistent with the molecular basis of BWS, which is primarily related to imprinting defects and epigenetic dysregulation at chromosome 11p15 rather than to sex-linked genetic mechanisms. Age-related data were inconsistently reported across studies, limiting the possibility of defining the typical timing of onset or diagnosis of hearing impairment. As a result, it remains unclear whether hearing loss in BWS is predominantly congenital, early-onset, or potentially progressive, highlighting the need for longitudinal audiological assessment in future investigations.

From an audiological perspective, the literature indicates a predominance of conductive over sensorineural hearing loss in patients with Beckwith–Wiedemann syndrome. Although etiological data are limited and not uniformly reported, several cases have been attributed to stapes fixation. In this regard, Takahashi et al. [6] suggested that “stapedial fixation might be due to maldevelopment of the stapedial lamina, which also originates from the second branchial arch”, supporting a possible developmental middle-ear anomaly as an underlying mechanism. At the same time, the occurrence of unilateral and severe forms within the conductive spectrum highlights the considerable heterogeneity of auditory manifestations in this population and suggests that different pathogenic mechanisms may coexist.

The literature consistently points to the predominance of conductive hearing loss (CHL) in Beckwith–Wiedemann syndrome, supported by reproducible anatomic–surgical findings—stapes fixation, malleus fixation, and incudostapedial abnormalities—that plausibly reflect second branchial arch developmental mechanisms; accordingly, CHL emerges as the most coherent and repeatable audiological phenotype reported to date.

By contrast, sensorineural hearing loss (SNHL) appears uncommon and is often described in historical reports with incomplete audiological methods, so overlooked conductive/mixed components or misclassification cannot be entirely excluded. In our cochlear implant case (Case 1), a comprehensive work-up (ABR thresholds, immittance, MRI, and high-resolution CT) confirmed true bilateral profound SNHL with normal middle-ear morphology, supporting a minor subgroup with genuine cochleo–neural involvement and reinforcing the need for standardized electrophysiology and, when indicated, imaging in BWS.

The available evidence supports the inclusion of comprehensive otologic and audiological evaluation within the multidisciplinary follow-up of patients with BWS and underscores the need for further studies aimed at clarifying the epidemiology, natural history, and etiopathogenetic mechanisms of hearing loss associated with this condition.

The three cases reported further emphasize the marked audiological heterogeneity associated with Beckwith–Wiedemann syndrome, encompassing both sensorineural and conductive forms of hearing loss with different degrees of severity and laterality. The rehabilitative approaches adopted—cochlear implantation, bone-conduction amplification, and conventional hearing aids—reflect the need for individualized management based on the underlying pathophysiology and functional impairment.

These clinical observations are consistent with the variability described in the available literature and further support the concept that hearing impairment in BWS may present with diverse clinical patterns requiring a comprehensive diagnostic work-up, including behavioral, electrophysiological, and, when indicated, neuroradiological assessment.

Overall, the heterogeneity of both audiological presentation and rehabilitative needs underscores the importance of a standardized, multidisciplinary otologic and audiological follow-up in patients with BWS.

## 5. Conclusions

Audiological involvement in Beckwith–Wiedemann syndrome (BWS) is increasingly recognized as a clinically relevant, though non-classical, feature. The systematic review of published reports highlights that the literature remains limited and fragmented, with heterogeneous audiological data and variable completeness of clinical and rehabilitative information. Despite these limitations, a predominance of conductive hearing loss over sensorineural deficits emerges, suggesting a possible role of ossicular malformations or embryologic middle-ear anomalies in the pathogenesis of hearing impairment in BWS.

The present study adds to the existing literature by providing detailed audiological data on three additional patients, including both surgical and rehabilitative management, with comprehensive documentation from diagnosis through follow-up. This represents the strength of the work, as previous reports often included incomplete audiological information and limited focus on management strategies.

Overall, these findings emphasize the importance of structured, multidisciplinary audiological follow-up initiated early, considering the potential evolution of auditory profiles over time and the frequent association of factors affecting communication and language development in BWS. These findings support the systematic inclusion of comprehensive audiological evaluation in the diagnostic and therapeutic pathways for patients with BWS, to enable early and targeted interventions and optimize long-term functional outcomes, while acknowledging that the currently available literature remains limited and warrants further systematic investigation.

## Figures and Tables

**Table 1:** Diagnostic clinical criteria for the Beckwith–Wiedemann spectrum (BWSp).

	Clinical Features
**Major findings** **(cardinal features)** **2 points**	Macroglossia Exomphalos Lateralized overgrowth (hemihypertrophy) Multifocal and/or bilateral Wilms tumor or nephroblastomatosis Hyperinsulinism (lasting >1 week and requiring escalated treatment) Pathology findings: adrenal cortex cytomegaly, placental mesenchymal dysplasia or pancreatic adenomatosis
**Minor findings** **(suggestive features)** **1 point**	Birthweight > 2SDS above the mean Facial naevus simplex Polyhydramnios and/or placentomegaly Ear creases and/or pits Transient hypoglycemia (lasting <1 week) Typical BWSp tumors (neuroblastoma, rhabdomyosarcoma, unilateral Wilms tumor, hepatoblastoma, adrenocortical carcinoma or phaeochromocytoma) Nephromegaly and/or hepatomegaly Umbilical hernia and/or diastasis recti

Interpretation: ≥4 points—clinical diagnosis of BWS; 2–3 points—molecular testing recommended; <2 points—BWS unlikely (Brioude et al., 2018) [2].

**Table 2:** Audiological characteristics, hearing loss severity, and genetic findings in patients with Beckwith–Wiedemann syndrome (BWSp) reported in the included studies.

Authors	No. of Patients	Type of Hearing Loss	Laterality	Degree of Hearing Loss	PTA (dB HL)	Genetic Findings
Daugbjerg et al., 1984 [5]	1	CHL	Bilateral	Mild	40	Not available
Takahashi et al., 1996 [6]	2	CHL	Bilateral	NA	NA	Not available
Schick et al., 1999 [7]	1	CHL	Bilateral	Moderate	55	No aberration in Chr 2
Hopsu et al., 2003 [8]	1	CHL	Bilateral	Moderate	70 (right), 25 (left)	Not available
Kantaputra et al., 2012 [9]	2	SNHL	Bilateral	Severe	NA	CDKN1C c.579delT (p.A193AfsX46)
			Bilateral	Mild	NA	
Camet et al., 2018 [4]	5	3 CHL	Bilateral	Mild	22	CDKN1C c.881delC (p.Pro294fs)
			Bilateral	Mild	26	
			SSD (right)	Mild	35	Hypermethylation of IC1.
		2 mixed/SNHL	Bilateral	Moderate	55	CDKN1C c.881delC (p.Pro294fs).
			Bilateral	Severe/profound	75	Hypomethylation of IC2.

PTA (dB): pure-tone average calculated across 500–1000–2000–4000 Hz. Abbreviations: CHL, conductive hearing loss; SNHL, sensorineural hearing loss; HL, hearing loss; NA, not available.

**Table 3:** Clinical features associated with Beckwith–Wiedemann syndrome (BWSp) reported in the included studies, grouped by clinical domain.

Clinical Domain	Clinical Features	No. of Patients	Authors
Craniofacial	Macroglossia	5	Daugbjerg et al.,1984 [5] Schick et al., 1999 [7] Kantaputra et al., 2012 [9]
	Cleft Palate	3	Kantaputra et al., 2012 [9]
	Maxillary hypoplasia	4	Kantaputra et al., 2012 [9] Daugbjerg et al.,1984 [5]
	Ear abnormalities	8	Daugbjerg et al.,1984 [5] Kantaputra et al., 2012 [9] Schick et al., 1999 [7] Kantaputra et al., 2012 [9]
Cognitive	Intellectual disability	1	Schick et al., 1999 [7]
	Delayed language	1	Schick et al., 1999 [7]
Gastrointestinal	Omphalocele	5	Daugbjerg et al.,1984 [5] Schick et al., 1999 [7] Kantaputra et al., 2012 [9]
Dermatological	Flame nevus *	2	Daugbjerg et al.,1984 [5] Schick et al., 1999 [7]

* “Flame nevus” corresponds to “nevus flammeus/port-wine stain” as described in the original reports.

**Table 4:** Middle-ear surgical findings and postoperative audiological outcomes in patients with Beckwith–Wiedemann syndrome (BWSp).

Authors	No. of Patients	Surgical Procedure	Additional Procedure	Intraoperative Finding	Audiological Outcome
Daugbjerg et al.,1984 [5]	1	Left tympanotomy	Stapedectomy	Stapedial fixation and cholesteatomatous keratin pearl	Hearing improved by 15–30 dB
Takahashi et al., 1996 [6]	2	Tympanotomy	Small fenestra stapedectomy	Stapedial fixation	Hearing improvements
Schick et al., 1999 [7]	1	Bilateral sequential tympanotomy	Small fenestra stapedectomy	Bony fixation of both mallei and ankylotic stapes	Conductive hearing loss was reduced on the right side to approximately 20 dB and on the left side to approximately 40 dB
Hopsu et al., 2003 [8]	1	Right tympanotomy	Partial stapedectomy	Stapedial fixation	Improved hearing level to 30 to 35 dB

“Tympanotomy” refers to an exploratory tympanotomy, i.e., surgical access to the middle ear for diagnostic assessment.

**Table 5:** A summary of audiological characteristics, interventions, and follow-up findings in the three patients with Beckwith–Wiedemann syndrome (BWSp) from our institution.

Patient	Sex	Age at First Evaluation	Type of Hearing Loss	Audiological Intervention	Outcome and Follow-Up
**1**	F	9 mo	Bilateral profound SNHL	Bilateral cochlear implant	Improvement in auditory perception; ongoing follow-up
**2**	M	15 y	Right severe CHL	BCD trial → planned implantation	Good functional improvement; on surgical waiting list
**3**	F	5 y	Bilateral moderate CHL	Hearing aids	Ongoing follow-up; language development monitored

Abbreviations: CHL, conductive hearing loss; SNHL, sensorineural hearing loss; BCD, bone conduction device.

## Data Availability

The data presented in this study are available from the corresponding author upon reasonable request.

## Abbreviations

The following abbreviations are used in this manuscript:

BWS	Beckwith–Widemann syndrome
HL	Hearing loss
CI	Cochlear implant
SNHL	Sensorineural hearing loss
CHL	Conductive hearing loss
BCD	Bone conduction device
PTA	Pure-tone average (dB)
dB	Decibel
AABR	Automated Auditory Brainstem Response
TEOAEs	Transient Evoked Otoacoustic Emissions
